# The Role of Low Dose Whole Body CT in the Detection of Progression of Patients with Smoldering Multiple Myeloma

**DOI:** 10.1038/s41408-020-00360-9

**Published:** 2020-09-25

**Authors:** Maria Gavriatopoulou, Andriani Βoultadaki, Vassilis Koutoulidis, Ioannis Ntanasis-Stathopoulos, Charis Bourgioti, Panagiotis Malandrakis, Despina Fotiou, Magdalini Migkou, Nikolaos Kanellias, Evangelos Eleutherakis-Papaiakovou, Efstathios Kastritis, Evangelos Terpos, Meletios A. Dimopoulos, Lia-Angela Moulopoulos

**Affiliations:** 1 Plasma Cell Dyscrasias Unit, Department of Clinical Therapeutics, School of Medicine, General Alexandra Hospital, National and Kapodistrian University of Athens, Athens, Greece; 2 1st Department of Radiology, School of Medicine, Aretaieion Hospital, National and Kapodistrian University of Athens, Athens, Greece

**Keywords:** Myeloma, Risk factors

## Abstract

Multiple myeloma (MM) is the second most common hematological malignancy, characterized by plasma cell bone marrow infiltration and end-organ involvement. Smoldering MM (SMM) is an intermediate clinical entity between MGUS and MM, with a risk of progression to symptomatic disease 10% per year. Bone disease is the most frequent symptom of MM, with ~90% of patients developing bone lesions throughout their disease course. Therefore, imaging plays a crucial role in diagnosis and management. Whole-body low-dose CT (WBLDCT) is widely available and has been incorporated in the latest diagnostic criteria of the IMWG. The purpose of this study was to evaluate the role of WBLDCT in the early identification of lesions in patients with SMM who progress solely with bone disease. In total, 100 asymptomatic patients were consecutively assessed with WBLDCT from July 2013 until March 2020 at baseline, 1-year after diagnosis and every 1 year thereafter. Ten percent of patients were identified as progressors with this single imaging modality. This is the first study to evaluate prospectively patients with SMM at different time points to identify early bone lesions related to MM evolution. Serial WBLDCT studies can identify early myeloma evolution and optimize disease monitoring and therapeutic strategies.

## Introduction

Multiple myeloma (MM) is the second most common hematological malignancy and it is characterized by bone marrow infiltration of monoclonal plasma cells and end-organ involvement^1^. Monoclonal gammopathy of undetermined significance (MGUS) is a premalignant plasma cell disorder, characterized by the absence of symptoms and organ involvement and limited plasma cell infiltration^2^. MGUS usually precedes MM evolution. The progression rate of MGUS to MM is ~1% per year^3^. Smoldering MM (SMM) is an intermediate clinical entity between MGUS and MM, with a risk of progression to symptomatic disease at 10% per year^3^. The diagnosis of MM is based on the International Myeloma Working Group (IMWG) criteria, which include: bone marrow plasma cell infiltration ≥10% and at least one CRAB feature (hypercalcemia, renal failure, anemia, and bone lesions), or ≥60% monoclonal plasma cell infiltration on bone marrow biopsy, serum involved to uninvolved free light chain ratio ≥100 or more than one >5 mm focal lesion at magnetic resonance imaging or a biopsy-proven bone or extramedullary plasmacytoma^4^.

Bone disease is the most frequent disease-related symptom of MM, with ~90% of patients developing bone lesions throughout their disease course. Therefore, imaging plays a crucial role both in diagnosis and management of these patients. Conventional radiography was traditionally considered as the standard of care, however the limited sensitivity in detecting osteolytic lesions has led to the use of more advanced imaging modalities. More specifically, at least 50% of trabecular bone must be destroyed for an osteolysis to be visible on plain radiography^5^. Imaging is crucial in discriminating MM from smoldering disease, since the presence of bone lesions establishes the diagnosis of active disease which requires treatment initiation^6^. Whole-body magnetic resonance imaging and positron emission tomography are more accurate in detecting MM induced bone disease, compared with plain radiography^7–10^. On the other hand, computed tomography (CT) was found to be the most sensitive modality in detecting small osteolytic bone lesions <5 mm^11,12^. Ionizing radiation exposure was one of the main limitations of this technique in the past. Technological advances have made possible low dose assessment of the entire skeleton with effective radiation doses comparable to those of a whole-body plain radiographic skeletal survey^13^. Furthermore, low dose CT is widely available and has a very short scan time. Therefore, whole-body low dose CT (WBLDCT) has been incorporated in the latest diagnostic criteria of the IMWG^4^. The purpose of this study was to evaluate the role of WBLDCT in the early identification of patients with asymptomatic MM who progress with bone disease only, and who require immediate antimyeloma treatment.

## Patients and methods

### Patients

Our study was approved by the local IRB and all patients provided written informed consent. All patients diagnosed with SMM based on the 2003 IMWG definition of SMM (serum M-protein of ≥3 g/dl and/or ≥10% bone marrow plasma cells with no evidence of end-organ damage i.e., hypercalcemia, renal insufficiency, anemia or bone lesions) were serially assessed with WBLDCT from July 2013 until March 2020 as part of our institutional workup^14^. The assessments were performed at baseline, 1-year post diagnosis and every 1 year thereafter. The patients enrolled in the study were those who had at least two consecutive CT assessments at the above described time points and were followed with hematologic, biochemical and immunological tests every 3 months for the first two years, and every 6 months thereafter.

### Whole-body low-dose CT

Between July 2013 and December 2017, all WBLDCT exams were performed on a 16-detector CT scanner (Philips Healthcare) using the following parameters: Tube voltage (kV)/time-current product (mAs) 120/60; collimation 16 × 1.5 mm; pitch 0.94; rotation time 0.50 s. Between January 2018 and March 2020, all WBLDCT exams were performed on a 64-detector CT scanner (Philips Healthcare) using the following parameters: Tube voltage (kV)/time-current product (mAs) 120/70; collimation 64 × 0.625 mm; pitch 1.00; rotation time 0.40 s. Both CT scanners have similar diagnostic sensitivity with regards to detecting myeloma-related bone lesions. All patients were scanned from the cranial vertex to the proximal tibia with the arms positioned either above the head with as little bending at the elbows as possible, or on top of the abdomen with the hands folded, and the humeri included in the field of view. Source images were reconstructed with a high-frequency reconstruction algorithm for detection of osteolytic lesions, and a smooth, soft tissue reconstruction algorithm for evaluation of the medullary cavities of the appendicular skeleton and for soft tissue assessment.

### Image analysis

All CT exams were evaluated in consensus by two experienced radiologists (L.A.M. and V.K. with 8 years of experience in interpreting WBLDCT scans of patients with plasma cell dyscrasias), who were blinded to the clinical and laboratory data. Image analysis was performed on a dedicated workstation using the Intellispace Portal diagnostic software (Philips Healthcare). The criteria for the identification of MM-related bone disease was the presence of at least one well-defined lytic lesion of the trabecular bone (≥5 mm) with no sclerotic margins, in line with established guidelines^4,15^. Hyperdense medullary lesions of the appendicular skeleton, and fractures (including vertebral compression fractures) were also recorded, but were not used for determining the presence of MM-related bone disease. Incidental findings, unrelated to myeloma, were also identified and recorded.

### Statistical analysis

The Wilcoxon rank test estimated differences between patients at different time points along with Student’s *t* test. The Spearman’s nonparametric correlation test determined the correlations between evaluated parameters. The Kaplan–Meier method was used to estimate time to progression (TTP), progression-free survival (PFS), and overall survival with differences compared by the two-sided log-rank test to identify potential prognostic factors. All *p* values were two sided and confidence intervals refer to 95% boundaries. The analyses were performed with the IBM SPSS version 25.0 statistical software.

## Results

We prospectively evaluated 100 patients with asymptomatic MM (median age at diagnosis 61 years range 40–86 years, 52 female/48 male) who underwent WBLDCT at the above described time points. Baseline characteristics of the patients are depicted in Table 1. Median number of WBLDCT exams performed was 2.5 (range 2–5); more specifically, three patients underwent five consecutive exams, nine patients four exams, 24 patients three exams, and the remaining 64 patients two consecutive WBCTs. The patients were stratified at diagnosis as low (none of the three risk factors), intermediate (one of the three risk factors), and high risk (two or three risk factors) according to the updated risk stratification criteria for SMM based on bone marrow infiltration above 20%, Mpeak above 2 gr/dl and FLC ratio above 20^16^. The relevant distribution for our patient group was 29%, 35%, and 31% for low, intermediate, and high-risk stage, respectively. During a median follow up of 57 (range 13–83) months, 31 patients have progressed (with either CRAB criteria and/or at least one myeloma defining event) and the distribution per prognostic risk stage was 3.2%, 32.3%, and 64.5% for low, intermediate, and high risk, respectively. Importantly, 10 of 31 patients progressed only with bone lesions that were identified on the scheduled WBLDCT as per protocol. None of the lesions was biopsied, since there was no ambiguous differential diagnosis. Patients who progressed only with myeloma-related bone disease did not have any other signs of progression to symptomatic MM. For this subgroup of patients, the distribution per intermediate and high-risk stage was 40% and 60% respectively. No significant differences were noted with regards to baseline hemoglobin, albumin, b2 microglobulin, FLCs, bone marrow infiltration, and other baseline patient characteristics between the bone and the other progressors (Table 1). The other 21 patients progressed with anemia (*n* = 4), bone marrow infiltration >60% (*n* = 2), abnormal free light chain ratio >100 (*n* = 3), while 12 patients progressed with more than one CRAB and/or myeloma defining events (*n* = 6 with two criteria, *n* = 4 with three criteria, *n* = 2 with four criteria).

**Table 1:** Baseline patient characteristics.

Variable	All (*n* = 100)	Bone-only progressors (*n* = 10)	Other progressors (*n* = 21)	*p* value (bone versus other)
Hb (g/dl)	12.9 (9.8–16)	13.4 (11.4–14.8)	12.6 (10.9–14.2)	0.245
WBC (×10^–3^)	6.2 (2.2–13.5)	5.9 (3.2–8.8)	6.0 (2.2–13.5)	1.000
PLTs (×10^−3^)	251 (94–686)	273 (178–451)	219 (146–582)	0.673
Cr (mg/dl)	0.8 (0.4–8)	0.8 (0.54–1.29)	0.73 (0.5–1.5)	0.245
Ca (mg/dl)	9.5 (7.39–11)	9.29 (8.5–10.6)	9.6 (8.9–10.6)	0.695
B2 microglobulin (mg/l)	2.2 (0.9–15)	2.21 (1.06–4.41)	2.33 (0.90–4.03)	1.000
LDH (U/l)	169 (103–325)	160 (103–221)	169 (110–274)	1.000
Alb (g/dl)	4.3 (3.2–5.3)	4.2 (3.7–4.8)	4 (3.2–4.7)	1.000
IgG (mg/dl)	1580 (358–5824)	1550 (626–4170)	1800 (420–5824)	1.000
IgA (mg/dl)	104 (5–4181)	60.7 (14–1336)	97.7 (22–1590)	1.000
IgM (mg/dl)	41.9 (4–369)	36.4 (12–171)	41 (4–205)	0.420
Mpeak (g/dl)	1.51 (0–4.9)	2.67 (0.86–3.66)	2.34 (0–4.94)	0.700
κFLC (mg/l)	21.9 (1.34–990)	29.6 (11.5–635)	31.8 (1.34–990)	1.000
λFLC (mg/l)	12.15 (1.08–9.88)	11.4 (5.2–760)	9.55 (1.08–988)	0.700
FLC ratio >8 (*n*, %)	33 (33.3)	5 (50)	11 (55)	0.796
BM infiltration (%)	20 (10–55)	22.5 (15–40)	35 (10–55)	0.260
Heavy chain (*n*)	97	10	19	0.266
IgG	23	6	16	
IgA	74	4	3	
Light chain only	3 (1κ, 2λ)	0	1	
Risk for progression^a^ (%)				0.773
Low	29	0	5	
Intermediate	36	40	25	
High risk	31	60	70	

Values are expressed as median (range), unless otherwise specified.

^a^According to risk stratification of smoldering multiple myeloma incorporating revised IMWG diagnostic criteria.

**Fig. 1:**
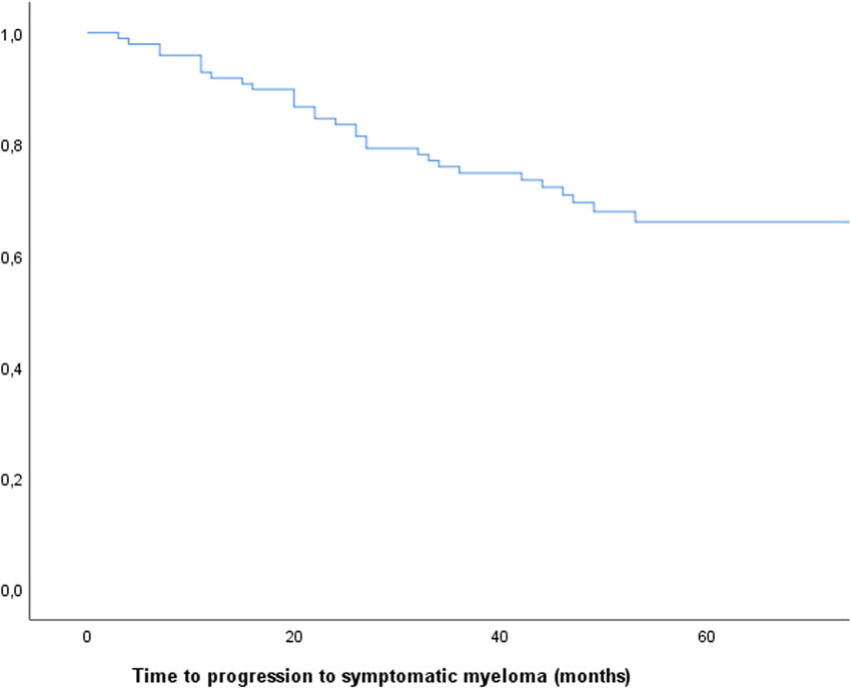
Kaplan–Meier curve for time to progression (TTP) from smoldering to symptomatic multiple myeloma. The median TTP from asymptomatic to symptomatic disease for all patients has not been reached, and was 8% at 1 year, 16% at 2 years, and 24% at 3 years.

**Fig. 2:**
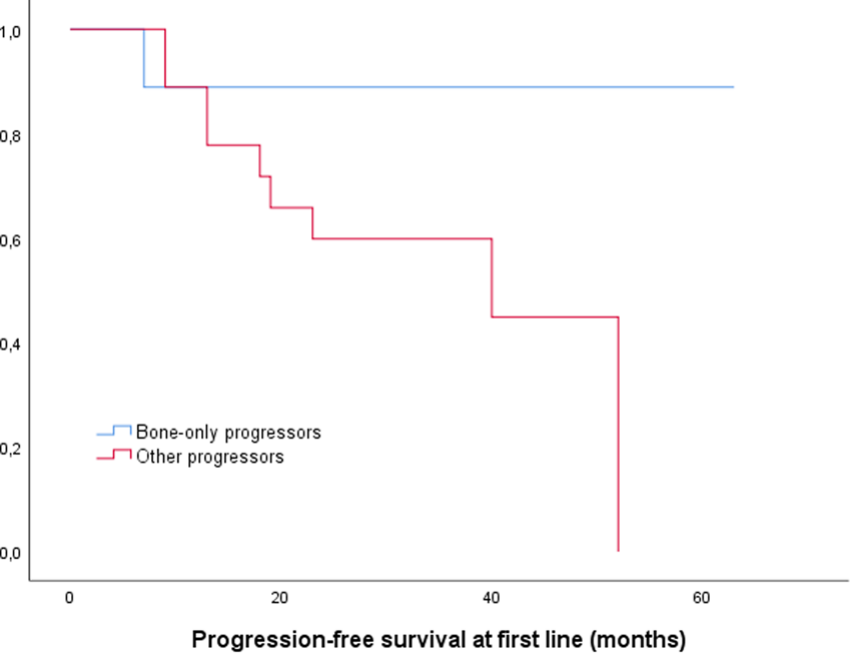
Kaplan–Meier curve for the progression-free survival (PFS) among patients with evolution to symptomatic multiple myeloma (*n* = 31). The median PFS has not been reached for bone progressors (eg patients with isolated bone disease at the time of progression, *n* = 10).

For the ten patients who progressed based on the results of WBLDCT, median age at baseline was 59 years (range 56–72). Median TTP from asymptomatic to symptomatic disease for all patients has not been reached, and was 8% at 1 year, 16% at 2 years, and 24% at 3 years (Fig. 1). Median TTP for those who actually progressed was 22 months (95% CI: 15.6–28.4). For the subgroup of patients who progressed with bone lesions only, the median TTP was also 22 months (95% CI: 3.4–40.6) and was not statistically different between the two progressor subgroups (progression only with bone disease versus all the others). At the time of disease progression, the bone marrow infiltration had increased significantly compared to baseline for patients that progressed only with bone disease (27% versus 44.4%, *p* = 0.036). The distribution per ISS stage at the time of progression was 48% for stage 1, 42% for stage 2, and 10% for stage 3 for all patients who progressed and it was 70% for stage 1 and 30% for stage 2 for those who progressed only with bone lesions. The R-ISS stage distribution was 50%, 46%, and 4% for stage 1, 2, and 3, respectively, for all patients who progressed, while for the bone progressors it was 60% and 40% for stage 1 and 2, respectively. Between the two subgroups there were no differences regarding the ISS and R-ISS distribution. All patients were initiated with antimyeloma treatment immediately post evolution to symptomatic disease. PFS for all 31 patients at first-line treatment was 52 months (95% CI: 34.5–69.5) and median PFS for bone progressors has not yet been reached (Fig. 2). Among the patients who progressed, 29 (94.5%) were alive at the time of the analysis. The two deaths that occurred were one related (progressive disease) and one unrelated to MM (cardiovascular event). Neither had progressed with isolated bone involvement.

### WBCT findings for patients who progressed only with bone lesions

**Fig. 3:**
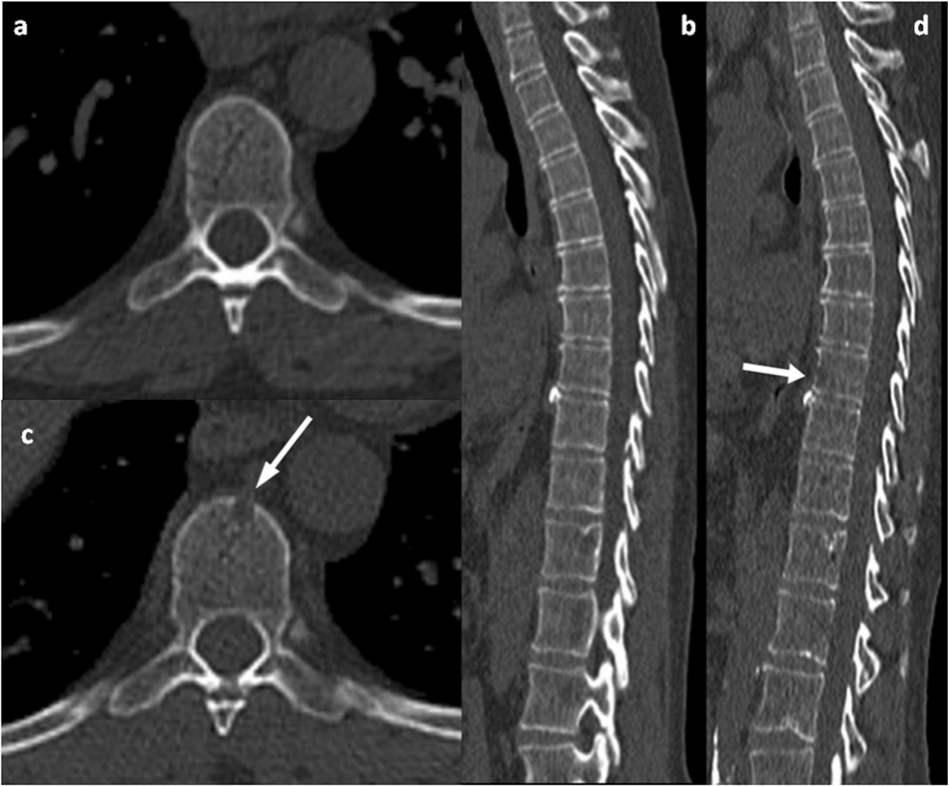
Case study of a bone-only progressor. A 58-year-old woman with smoldering myeloma. Axial WBLDCT image at the level of T9 (**a**) and sagittalreconstruction (**b**) shows no osteolysis. Corresponding axial (**c**) and sagittal reconstruction (**d**) images from a WBLDCT study performed 2 years later, show single subtle, small osteolysis with cortical erosion at T9 (arrow in **c** and **d**). The patient had no other signs of symptomatic disease.

Among the patients who progressed based only on WBLDCT findings, two patients presented with skull lesions, five with spine lesions mainly thoracic, one with a single rib lesion with associated fracture, one with an iliac lesion, one with pelvic lesions, and one with extensive bone disease (Fig. 3). However, none of the patients reported symptoms of bone disease (such as pain) or hypercalcemia at the time of evaluation with WBLDCT.

### Incidentalomas

During the review of the CT scans several clinically significant and non-significant findings were identified in 42 patients (42%): gallstones (*n* = 3), adrenal gland incidentalomas (*n* = 6), uterine fibromas (*n* = 5), nephrolithiasis (*n* = 5), polycystic kidneys (*n* = 1), large kidney cysts (*n* = 2), breast fibro adenomas (*n* = 4), abdominal lymphadenopathy (*n* = 1), diverticulosis (*n* = 4), femoral osteonecrosis (*n* = 1), lung nodules (*n* = 3), ovarian cysts (*n* = 2), spine hemangiomas (*n* = 8), Paget disease (*n* = 1), thyroid nodules (*n* = 2), thoracic and abdominal aortic aneurysms (*n* = 2), brain arachnoid cysts (*n* = 2), lipomas (*n* = 2), psoas aplasia (*n* = 1). The above-mentioned findings were not reported at patients’ medical history. Regarding the clinically significant findings, patients were guided accordingly for further diagnostic evaluations and therapeutic interventions.

## Discussion

Our study is the first one to evaluate consecutive SMM patients with WBLDCT at different time points to identify early bone lesions related to MM evolution; WBLDCT provides important information for disease monitoring and detection of significant findings, therefore improving both diagnosis and management of these patients. Although the survival of patients with MM has improved significantly the past decade following the incorporation of novel treatment agents, it remains an incurable disease^17–19^. Therefore, sensitive techniques for early diagnosis and accurate staging are more than necessary. Imaging plays a critical role for the early detection of symptomatic disease, since the presence of bone disease indicates that treatment initiation is required^6^. MM is preceded by MGUS and SMM, precursor modalities that are characterized by the absence of bone lesions, symptoms, and organ impairment. Based on that, it is of high importance to limit the possibility of bone involvement to rule out disease progression. Moreover, bone disease is a major cause of mortality and is correlated with worse prognosis^4^. WBLDCT has been increasingly used in the screening of patients with plasma cell dyscrasias, since it is considered one of the most accurate techniques in detecting MM-related bone disease and has replaced whole body plain radiography^11,12,20–27^. In one study, WBCT assessed therapy response in patients with MM and was proven more reliable than conventional follow-up^21^. Another study indicated that WBCT was very reliable for lesion detection and MM staging^27^. Simeone et al. in a retrospective study reported that WBLDCT led to a change in management in 28% of patients with MM or its precursor states^28^. However, the role of consecutive exams at specific time points for early identification of new bone lesions in patients with SMM has not been investigated. The aim of our study was to assess the exact role of WBLDCT in the diagnosis and management of patients with SMM and identify whether some patients progress only with bone disease identified with this specific modality. Mean effective radiation dose of our protocol was comparable to other low-dose WBCT protocols reported in the literature^20,25,27^.

In total, 100 patients with SMM were evaluated prospectively in our study and were followed periodically with WBLDCT at the described time points of the protocol. Our study showed, as expected, that the majority of patients who progressed from asymptomatic to symptomatic disease were characterized at diagnosis as intermediate or high risk. In total, 31/100 patients (31%) progressed but, most importantly, ten (10%) patients were identified as progressors only because of bone lesions on WBLDCT, with no other CRAB criteria or myeloma defining events. Another important finding was the PFS difference between those who progressed for all reasons and those who progressed with bone lesions only. Although the patient number is rather small, a potential trend has emerged. This may be due to the early diagnosis and intervention and the avoidance of potential risks and comorbidities that accompany a late diagnosis (e.g. severe anemia, infections, fractures, renal impairment). The hypothesis that early treatment initiation will significantly improve the disease outcomes even in the asymptomatic setting has been investigated for many years^29^. The main therapeutic strategies focus either on prevention of progression or definitive therapy in order to eradicate the disease at an early state and achieve cure^30^. Since SMM is a heterogeneous biological entity, it is of high importance to identify the early myeloma patients and the clonal evolution. This way, an early therapeutic approach might be more effective before further immune dysregulation. More studies are required to clarify the exact role of early interventions. Although it is not yet proven, there is a hypothesis that early myeloma is genetically less aggressive, and with optimal treatment, a subgroup of patients could be cured; this may be the case with this subgroup of patients who are identified early in the disease course with bone lesions only.

Finally, comprehensive review of WBLDCT images led to identification of incidental findings in a large number of patients; some of these required further investigation and treatment. Our results were anticipated and comparable to a recent report which evaluated the role of WBCT in prevention and early diagnosis in general population and demonstrated that the high mortality associated with cancer, cardiovascular, and other diseases may be reduced. In this study which evaluated retrospectively 6516 subjects who underwent WBCT the most common WBCT findings in asymptomatic subjects were benign. However, this imaging technique identified an important number of significant and precocious findings^31^.

Among the strengths of our study is the prospective study design and the long term follow up. Furthermore, the study included a large number of patients using a dedicated protocol for the management of patients with monoclonal gammopathies and the majority of the patients underwent several imaging assessments. Moreover, both patient management and WBLDCT evaluation were carried out in expert myeloma centers to assure close monitoring of the patients and high-quality imaging data. The main limitation of the study is the relatively small patient number in subgroup analysis. Another possible limitation may pertain to the lack of cytogenetic studies at the time of SMM diagnosis which could offer more prognostic and valuable insight. Furthermore, it has to be noted that none of the newly identified myeloma-related bone lesions were biopsied in order to confirm the diagnosis of progressing symptomatic myeloma. However, there was no ambiguous differential diagnosis and all patients have been followed in our department and none of them had any signs or symptoms of an alternative diagnosis. The updated imaging guidelines for MM by the IMWG support the biopsy in case of uncertainty, especially for patients with MGUS.^32^

In conclusion, our strategy allowed early detection of bone lesions in 10% of SMM patients who were immediately initiated with antimyeloma treatment to avoid further myeloma-related complications. Serial low-dose WBCT imaging studies can identify early myeloma evolution to symptomatic disease and optimize the disease monitoring along with our therapeutic strategy, in accordance to the IMWG imaging guidelines.^32^
